# Pre-operative Embolization of a Cerebellar Hemangioblastoma Using Polyvinyl Alcohol (PVA) and Target Tetra 360 Detachable Coil

**DOI:** 10.7759/cureus.56891

**Published:** 2024-03-25

**Authors:** Alice S Wang, John C Murnin, James Wiginton IV, Konstantin Tchalukov, Charles E Stout, Jason Duong, Raed Sweiss

**Affiliations:** 1 Neurosurgery, Riverside University Health System Medical Center, Moreno Valley, USA; 2 Medicine, Burrell College of Osteopathic Medicine, Las Cruces, USA; 3 Radiology, Riverside University Health System Medical Center, Moreno Valley, USA; 4 Neurointerventional Radiology, Riverside University Health System Medical Center, Moreno Valley, USA; 5 Neurosurgery, Arrowhead Regional Medical Center, Colton, USA

**Keywords:** target tetra, coil, preoperative embolization, pva, cerebellar hemangioblastoma

## Abstract

Due to its hypervascularity, hemangioblastoma, a rare primary central nervous system intracranial tumor, has been treated with pre-operative embolization prior to surgical resection. Here, we describe a case treated as such. A 37-year-old male presented with worsening chronic headache and right ear tinnitus was found to have a hypervascular, heterogeneous right cerebellar lesion suspicious for arteriovenous malformation or hemangioblastoma. He underwent polyvinyl alcohol (PVA) and Target Tetra 360 (Fremont, CA: Stryker Neurovascular) detachable coil embolization followed by complete tumor resection. Pathology was consistent with hemangioblastoma. He presented with complete resolution of his symptoms immediately post-operatively and at a two-week follow-up. Our case highlighted the importance of pre-operative embolization to help achieve complete tumor resection which is considered curative in the treatment of hypervascular hemangioblastoma. The Target Tetra 360 detachable coil embolization is another material that can be considered.

## Introduction

Hemangioblastoma presents only 2% of all intracranial neoplasms and constitutes 7-12% of posterior fossa tumors. It is a WHO Grade 1 benign central nervous system tumor [1]. Approximately 25% of hemangioblastomas are a part of the von Hippel-Lindau (VHL) disease and approximately 75% are sporadic [2]. Clinical presentations include headache, ataxia, tinnitus, dizziness, vertigo, reduced hearing/deafness, oculomotor nerve dysfunction, nystagmus, dysphagia, motor weakness, or sensory deficits [3]. Radiographic patterns of hemangioblastoma on computed tomography (CT) and magnetic resonance imaging (MRI) include pure cyst (8%), mural nodule (35%), cyst with wall enhancement (6%), cystic nodule (6%), solid with cyst (12%), and solid (33%) [4]. Treatment modalities for symptomatic hemangioblastomas include surgical resection, stereotactic radiosurgery (SRS), radiation therapy (RT), and medication therapy, whereas asymptomatic hemangioblastomas can be observed.

Hypervascularity of the hemangioblastoma makes surgical resection challenging due to the major risk of massive intraoperative bleeding, which can prevent complete tumor resection [5]. Therefore, pre-operative embolization has been used. Sultan et al. reported 10 patients who received pre-operative embolization prior to surgical resection of the tumor. The materials used for embolization include polyvinyl alcohol (PVA), n-butyl cyanoacrylate (NBCA), and onyx. Total endovascular occlusion was achieved in six patients, near total occlusion in three patients, and incomplete occlusion in one patient. Nine patients had gross total resection and only one patient had onyx had subtotal resection. The total blood loss ranged from 130 to 850 mL [6]. Recently, the Target Tetra 360 detachable coil (Fremont, CA: Stryker Neurovascular) received FDA clearance in December 2022 for use in (1) intracranial aneurysms, (2) other neurovascular abnormalities such as arteriovenous malformations and arteriovenous fistulae, and (3) arterial and venous embolization in the peripheral vasculature [7]. Here, we describe an off-label use of the Target Tetra 360 detachable coil device along with PVA in pre-operative embolization that assisted in the complete resection of a hemangioblastoma with complete resolution of the patient’s clinical symptoms.

## Case presentation

**Figure 1:**
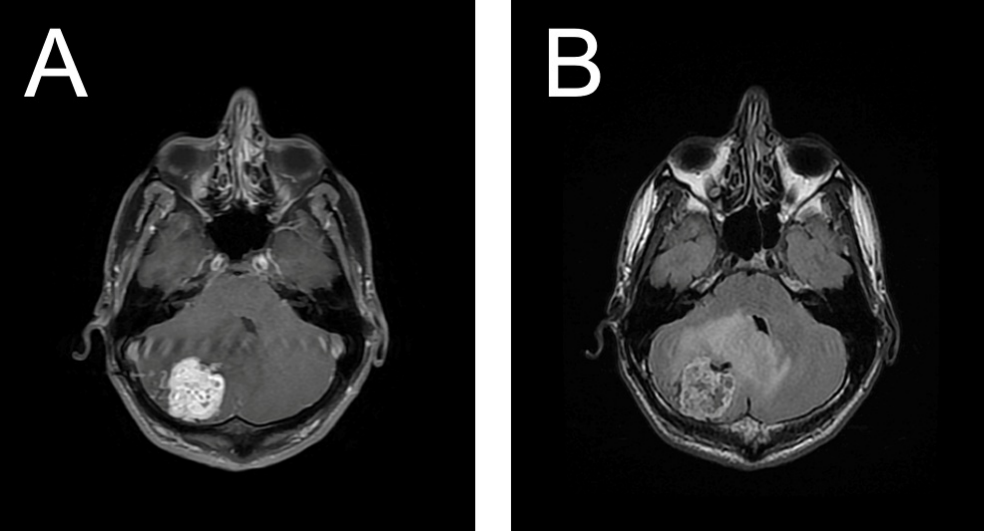
Pre-operative MRI shows a right cerebellar mass with associated vasogenic edema. (A) Axial T1-weighted contrast-enhanced image demonstrates avidly enhancing mass in the right posterior cerebellar hemisphere abutting the pial surface, with mass effect causing partial effacement of the fourth ventricle, consistent with hemangioblastoma. (B) Axial T2-FLAIR weighted image demonstrates a heterogenous, intraparenchymal mass in the right posterior cerebellar hemisphere abutting the pial surface, with perilesional cysts and intratumoral flow voids. There is marked vasogenic edema with partial effacement of the fourth ventricle.

**Figure 2:**
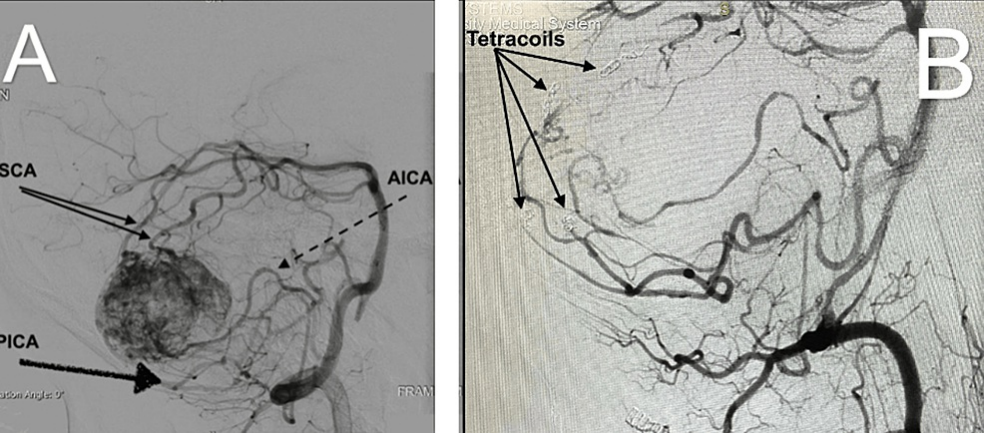
Cerebral angiogram shows multiple pedicles feeding the right cerebellar mass. (A) Diagnostic cerebral angiography via left vertebral artery shows multiple posterior circulation arteries feeding the tumor. (B) Post-coil embolization shows a total of four tetra coils placed, two in two SCA pedicles and two in PICA pedicles. SCA: superior cerebellar artery; AICA: anterior inferior cerebellar artery; PICA: posterior inferior cerebellar artery

A 37-year-old male presented at an outside hospital with a complaint of headache for one year that had been progressively worsening. The headache disturbed his sleep and was worse in the morning and with certain positions (bending forward and bending his neck). He also presented with right ear “whooshing” sound and worsening tinnitus which resulted in diminished hearing. CT head showed a right heterogenous cerebellar mass lesion and effacement of the fourth ventricle outflow with early ventriculomegaly and dilated temporal horns. Decadron 6 mg every 6 h was started to decrease vasogenic edema which helped relieve his headache and tinnitus. MRI brain T1 weighted contrast and T2-fluid-attenuated inversion recovery (FLAIR) showed an enhancing right cerebellar lesion of 3.5×2.7×3.3 cm suspicious for arteriovenous malformation (AVM) or hemangioblastoma and compression of the fourth ventricle with associated mild hydrocephalus and venous engorgement in the cavernous sinuses and veins at the skull base (Figures 1A, 1B). CT chest/abdomen/pelvis was negative for metastasis. CT angiography (CTA) head was most consistent with a large AVM with a nidus of 3.5×3.1×3.2 cm. Diagnostic cerebral angiogram (DCA) showed a hypervascular right cerebellar tumor with two right superior cerebellar artery (SCA) pedicles, one right anterior inferior cerebellar artery (AICA) pedicle, and three right posterior inferior cerebellar artery (PICA) pedicles (Figures 2A, 2B). The patient underwent successful PVA (Contour; Marlborough, MA: Boston Scientific) embolization (255-350 micron PVA particles) and Target Tetra 360 detachable coil embolization of the following amenable pedicles: two right SCA (one 2×6 cm coil for each pedicle) and two right PICA pedicles (one pedicle treated with 2×6 cm coil and the other with 2×4.5 cm coil) [8]. The patient tolerated the procedure well. There were no post-procedural complications.

**Figure 3:**
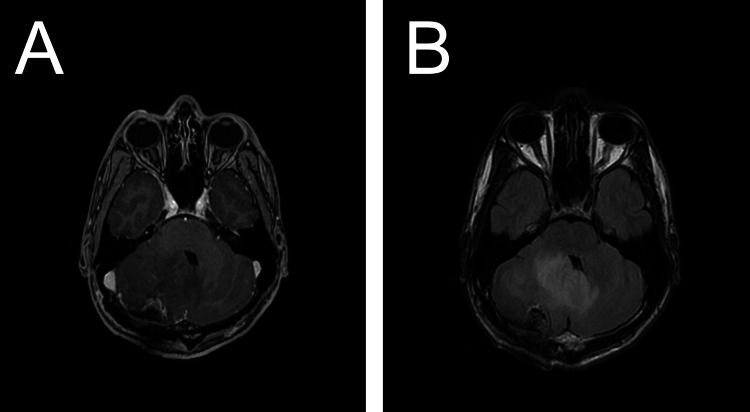
Post-operative MRI shows gross total resection of the right cerebellar mass with pathology consistent with hemangioblastoma. (A) Post-operative axial T1-weighted contrast-enhanced image demonstrates a resection cavity lined by thin, linear enhancement consistent with post-operative changes. (B) Post-operative axial T2-FLAIR weighted image demonstrates resection cavity with persistent mass effect. FLAIR: fluid-attenuated inversion recovery

The next day, the patient underwent right suboccipital craniectomy for resection of the cerebellar mass with stereotactic and ultrasonic guidance. The tumor was removed after 360-degree revascularization. Post-operative MRI T1-weighted contrast and T2-FLAIR showed gross total resection and decreased vasogenic edema (Figures 3A, 3B). There was minimal blood loss during the resection (45 mL) and no blood transfusion was required. Patient tolerated the surgery well. Pathology revealed central nervous system (CNS) WHO Grade 1 hemangioblastoma (epithelial membrane antigen {EMA} negative, inhibin positive). Patient was neurologically intact and was discharged from the hospital on post-operative day four. At two-week follow-up, the patient remained symptom-free. The incision was well healed. Patient is undergoing VHL workup.

## Discussion

Hemangioblastomas are rare, benign, highly vascular tumors that can arise from the CNS [9]. They are comprised of neoplastic stromal cells in a bed of excess vascularization, normal endothelial cells, pericytes, and mast cells. The most common location is in the cerebellum, and cerebellar hemangioblastomas are categorized into four distinct types. Type 1, 5% of posterior fossa hemangioblastomas, is described as a simple cyst with no visible nodules. Type 2, 60%, is a cyst with a mural nodule. Type 3, 26%, is a solid tumor (or tumors) with no cystic component. Type 4, 9%, is a solid tumor (or tumors) with small internal cysts [10].

Not all cerebellar hemangioblastomas cause symptoms. In asymptomatic hemangioblastomas, usually smaller in size compared to those of symptomatic hemangioblastomas, watchful waiting is the preferred course of action. They may remain stable for many years [11,12]. The average growth rate of a spontaneous hemangioblastoma is 1.79 cm^3 ^per year (19% per year) [13]. Over time, progressive growth of the tumor is associated with increased mass effect on its neighboring structures and, eventually, the patient may experience symptoms such as headache, ataxia, tinnitus, dizziness, vertigo, reduced hearing/deafness, cerebellar ataxia, oculomotor nerve dysfunction, nystagmus, dysphagia, motor weakness, or sensory deficits [3]. Due to its location, cerebellar hemangioblastomas can lead to blockage of the ventricular outflow tracts either through compression or hemorrhage causing obstructive hydrocephalus, which if not treated in a timely manner can result in significant morbidity and mortality. For example, in extreme cases, herniation and brain stem compression have been observed [14].

Surgical resection is the mainstay of treatment and complete surgical resection of the tumor is curative. However, massive intraoperative bleeding from the tumor bed can obstruct the surgeon’s view and prevent complete tumor resection [5]. Therefore, the purpose of pre-operative embolization is to reduce the total amount of blood flow to the tumor to minimize the risk of excessive bleeding during surgery. PVA, NBCA, and Onyx are embolization materials that have been used to help achieve occlusion of arterial feeders. This helped minimize blood loss and resulted in complete tumor resection in 90% of the cases [6].

In this case, the patient was symptomatic from the tumor. The hemangioblastoma on CT and MRI mimics an arteriovenous malformation, which is a cerebrovascular pathology for an on-labeled use of the Target Tetra 360 detachable coil device [7]. On DCA, the findings are more consistent with a neoplasm, such as a hemangioblastoma, than an AVM. We considered pre-operative embolization of the tumor. In patients with hemangioblastomas, PVA has been demonstrated to be helpful in lowering blood loss during surgery and enhancing surgical outcomes [15]. They are combined with a foaming agent and a hardening agent, such as formaldehyde, to create a solid, sponge-like, embolic agent with an irregular shape. When introduced, the sponge-like characteristics of the PVA particles enable them to conform to a cavity, expand, and harden [16]. PVA particles are available in various sizes and are useful as they are inert, water-insoluble, and biocompatible [17]. Moreover, PVA can induce a pro-thrombotic inflammatory response, which can aid in the devascularization of the hemangioblastoma [18]. The use of PVA will decrease the blood flow to the tumor, but we hypothesized that the use of PVA and adjunct Target Tetra 360 detachable coil will further reduce the blood flow to the tumor which is critical in this hypervascular hemangioblastoma to prevent excessive intraoperative bleeding. We decided to use the Target Tetra 360 detachable coil device because it has a new secondary shape, modified stretch-resistant fiber configuration, and a modified link design that provides strong shape stability while also being a very soft coil [7]. Therefore, the patient received PVA and Target Tetra 360 detachable coil embolization 24 hours prior to surgery. Indeed, the tumor was removed en bloc with minimal blood loss (<50 mL). Our case demonstrates that a coiling device may be a useful adjunct therapy in pre-operative embolization. Complete tumor resection is considered curative and we achieved this in our case, reducing the surgical risks of bleeding (with embolization), infection (shorter intraoperative time), and damage to adjacent structures (good visualization of the surgical cavity) [19-21].

There are several other treatment modalities for symptomatic cerebellar hemangioblastoma as follows: stereotactic radiosurgery, radiation therapy, and medication therapy. Success has been shown with combinations of radiation therapy after incomplete tumor excision [22].

SRS is an alternative to surgical resection in solid hemangioblastomas (Type 3). While it is not commonly used today, it has been shown to be an exciting alternative with promising results [20-22]. SRS is not commonly used in cystic tumors because radiation can temporarily increase vascular permeability, resulting in swelling around the tumor and the development of cysts [23-25]. A possible solution to this problem is aspiration of the cystic component prior to SRS [19]. If this method is employed, Goyal et al. conclude SRS would be viable for Types 3 and 4 and selective cases of Type 2 hemangioblastomas [26]. Moss et al. and Goyal et al. also agreed that SRS can be beneficial in treating asymptomatic hemangioblastomas <3 cm in size. An additional consideration in the use of SRS is the risk of radiation toxicity causing radiation necrosis. In instances of multiple tumors with overlapping radiation fields, this can be of particular concern [21,26].

RT is a treatment option typically reserved for tumors not amenable to surgical intervention, as well as recurrent and/or multiple lesions. Fractionated External Beam Radiation (EBRT) is suitable for situations where resection or SRS is not feasible or practical. This includes cases where there are multiple tumors with widespread intracranial or spinal cord involvement, tumors located near critical structures like the brainstem, and tumors that are too large or complicated to be safely treated with surgery or SRS [22]. A relationship between radiation dose and five-year overall survival has been proposed by Smalley et. al. A 50 Gray (Gy) has been used as a cutoff for previous studies, showing >50 Gy EBRT to have a five-year overall survival (OS) of 24% greater than if <50 Gy EBRT is delivered [26]. Side effects to consider when implementing EBRT include headache, nausea, vomiting, fatigue, skin irritation, seizures, cognitive changes, memory problems, and radiation-induced necrosis. Studies conducted on the use of EBRT have found combined (VHL and non-VHL) five-year OS rates between 69% and 85% and a 10-year OS of 30% [22,27-28]. There is little data on the application of EBRT for cerebellar hemangioblastoma due to more efficacious interventions existing in microsurgical resection and SRS.

Pharmacotherapy is a treatment alternative when surgical resection or SRS is not possible due to tumor location or complexity. Vascular endothelial growth factor (VEGF), a pro-angiogenic signal protein, has been shown to play a key role in the formation of blood vessels. VEGF is upregulated in the stromal cells of hemangioblastomas, which may indicate a possible mechanism for both tumor development and potential pharmacologic treatment [29]. Bevacizumab, a monoclonal antibody that inhibits VEGF-A by binding to VEGF and inhibiting its interaction with the VEGF receptor, has been suggested as a potential pharmacologic therapy for hemangioblastoma [30]. To the extent of our knowledge, there are no case reports of its use in spontaneous cerebellar hemangioblastoma. However, one case report successfully showed regression and clinical improvement in a patient with unresectable spinal cord hemangioblastoma [31]. EGFR tyrosine kinase inhibitors, interferon-alfa-2a, anti-angiogenic agents, and VEGFR2 inhibitors (SU5146) have shown suboptimal outcomes [32-35].

Though total surgical resection is widely regarded as curative, studies do show recurrence rates of 5-17%, though it should be noted that complete resection may only be achieved in 86-93% of cases [36,37]. Cases where subtotal resection is achieved are often associated with bleeding risks associated with further removal, tumor location, or infiltration of surrounding brain parenchyma. This patient had no such issues and total resection was achieved. They will be monitored for recurrence and were advised to undergo a workup for VHL, given its autosomal dominant inheritance pattern and association with 25% of cerebellar hemangioblastomas.

One of the strengths of this study is that this research is the first to show the safe and efficient use of a Target Tetra detachable coil in the resection of a cerebellar hemangioblastoma with multiple pedicles. This study provides insight into the potential use of Target Tetra coils in the treatment of cerebellar hemangioblastoma, which could benefit future patients. Additionally, the authors have provided a thorough explanation of the procedure and tools used, which may be helpful for those thinking about using Target Tetra in similar cases.

When interpreting the results of this case study, it is important to consider several limitations. This case report contains data collected from one patient, and as such, it is possible that the findings are not generalizable to larger populations or patients with different risk factors. Additionally, the patient's VHL status is currently undetermined, and while they did not exhibit typical features associated with VHL, there is a possibility that they have an undiagnosed VHL syndrome. The etiology of spontaneous cerebellar hemangioblastoma is not yet fully understood, but it is possible environmental factors could play a role. To this end, the study does not provide data on potential confounding variables such as socioeconomic status, lifestyle habits, smoking, or alcohol use, which may or may not influence the development of hemangioblastoma. To the best of the authors' knowledge, there are currently zero medical case reports or studies in the medical literature on the use of this product in cerebellar hemangioblastoma. Future research could aim to include larger patient pools to enhance the generalizability of the results. Future research could also investigate the efficacy of Target Tetra both as a stand-alone device and in conjunction with other devices for pre-operative embolization in treating cerebellar hemangioblastoma. By exploring these questions, researchers may be able to gain a better understanding of when to utilize Target Tetra in the treatment of cerebellar hemangioblastoma.

## Conclusions

In conclusion, cerebellar hemangioblastoma is a rare intracranial neoplasm that can be treated with surgery, radiation, medications, or a combination of the above. Pre-operative embolization helps reduce the risk of excessive intraoperative bleeding. Here, we describe a case using PVA and adjunct Target Tetra 360 detachable coil that allowed for complete tumor resection with complete resolution of symptoms. Our case demonstrates that coiling can be a useful adjunct therapy in the pre-operative embolization in the treatment of hemangioblastoma. More studies are needed to investigate its usefulness in treating other brain tumors.
